# Transcontinental Spread of HPAI H5N1 from South America to Antarctica via Avian Vectors

**DOI:** 10.3390/v17101365

**Published:** 2025-10-13

**Authors:** Ruifeng Xu, Minhao Gao, Nailou Zhang, Zhenhua Wei, Zheng Wang, Lei Zhang, Yang Liu, Zhenhua Zheng, Liulin Chen, Haitao Ding, Wei Wang

**Affiliations:** 1 Wuhan Institute of Virology, Chinese Academy of Sciences, Wuhan 430071, China; xurf@wh.iov.cn (R.X.); Zhangnailou@wh.iov.cn (N.Z.); Zhanglei@wh.iov.cn (L.Z.); liuyang@wh.iov.cn (Y.L.); zhengzh@wh.iov.cn (Z.Z.); 2 Polar Research Institute of China, Shanghai 200136, China; gaominhao@pric.org.cn (M.G.); weizhenhua_ah@163.com (Z.W.); wangzheng01@pric.org.cn (Z.W.); chenliulin@pric.org.cn (L.C.); dinghaitao@pric.org.cn (H.D.)

**Keywords:** H5N1, Antarctica, next-generation sequencing, Avian

## Abstract

During China’s 41st Antarctic research expedition, samples were collected from wildlife on the Fildes Peninsula, South Shetland Islands, Antarctica. Real-time RT-PCR screening confirmed H5N1 positivity, representing the first identification of the virus in brown skuas on the Fildes Peninsula. Whole-genome sequences obtained from positive samples via next-generation sequencing were subjected to phylogenetic and phylogeographic analyses. The results revealed that these Antarctic strains are most closely related to H5N1 viruses circulating in South America, particularly from Peru and Chile, suggesting a likely introduction via avian migration routes. Furthermore, a unique 17-amino-acid deletion was identified in the stalk region of the neuraminidase (NA) gene, which is uncommon among globally sampled clade 2.3.4.4b variants. This study confirms the arrival of HPAI H5N1 in the Antarctic continent and underscores the necessity for enhanced surveillance to understand the viral ecology and potential risks within this unique ecosystem.

## 1. Introduction

Highly Pathogenic Avian Influenza (HPAI) H5N1 viruses originating from the A/Goose/Guangdong/1/96 (Gs/GD) lineage have demonstrated rapid genomic evolution and remarkable cross-species transmission capabilities. Clade 2.3.4.4b outbreaks have intensified significantly since 2020, spreading rapidly across Asia, Europe, Africa, North America, and South America, with substantial impacts on poultry and wildlife [1]. Notably, this clade has demonstrated unprecedented adaptive evolution in mammalian hosts, with confirmed infections in dairy cattle and domestic cats on farms [2], as well as mass mortality events in southern elephant seals [3]. Human infections have also been documented [4], though sustained human-to-human transmission has not been observed.

The Antarctic region encompasses the Antarctic ice shelves, surrounding waters, and all island territories south of the Antarctic Convergence (Antarctic Polar Front), a marine boundary where cold Antarctic waters meet the warmer sub-Antarctic waters [5]. This region supports unique ecosystems that serve as critical habitats for numerous avian and marine mammal species. Despite their geographical isolation, wildlife, such as brown skuas (*Stercorarius antarcticus*), southern giant petrels (*Macronectes giganteus*), the Southern Elephant Seal (*Mirounga leonina*), and the Antarctic Fur Seal (*Arctocephalus gazella*), regularly breed in Antarctica but partially migrate to the coasts of Chile and Argentina during winter. Growing evidence suggests that viruses from South America may be spreading southward to Antarctica. In 2023, HPAI H5N1 was detected in sub-Antarctic regions including South Georgia (54°15′ S, 36°45′ W) and the Falkland Islands (51°42′ S, 57°51′ W) [6]. By 2024, the virus had reached James Ross Island (64°10′ S, 57°45′ W) in Antarctica, where it was identified in brown skuas, though no signs of HPAI were observed in wildlife on the Fildes Peninsula [7].

## 2. Materials and Methods

### 2.1. Sample Collection

During the 41st Chinese Antarctic Research Expedition, field investigations were conducted across the Fildes Peninsula and surrounding areas from December 2024 to February 2025. Expedition members wearing full personal protective equipment collected influenza samples from both live animals and atypical mortality cases using standardized protocols for sterilization, disinfection, and hazardous waste disposal. The spatial distribution of sampling sites was mapped using Google Earth. A total of 11 biological samples were obtained, including oropharyngeal swabs, cloacal swabs, fecal samples, and brain tissue specimens. All samples were preserved in cryotubes containing 1 mL RNA later and stored at 4 °C after collection and preserved at −80 °C.

### 2.2. qPCR

For viral genetic testing, we utilized a portable real-time fluorescence PCR system and Influenza A Virus with H5/H7 Subtype Nucleic Acid Detection Kit (PCR-fluorescent probe method) (Zhongkeshengyi science and technology Co., Ltd., Beijing, China), following the manufacturer’s instructions for avian influenza virus screening. Samples with Ct values ≤35, accompanied by a characteristic S-shaped amplification curve, were identified as avian influenza virus-positive.

### 2.3. RNA Extraction

The processed samples were subjected to RNA extraction using the MiniBEST Viral RNA/DNA Extraction Kit Ver. 5.0 (Takara, Osaka, Japan) following the manufacturer’s protocol. The extracted RNA was immediately stored at −80 °C until downstream molecular analysis. For each extraction procedure, RNase-free water was included as a negative control.

### 2.4. Next-Generation Sequencing

Influenza virus-positive samples identified via PCR-fluorescent probe assay were selected for Next-generation sequencing (NGS) to analyze genomic characteristics. Total RNA libraries were constructed using the VAHTS Universal V8 RNA-seq Library Prep Kit for Illumina (Vazyme, Nanjing, China) in accordance with the manufacturer’s protocol. Approximately 1 μg of total RNA was reverse-transcribed into cDNA, followed by fragmentation, adapter ligation, PCR amplification, and purification. Library quality control was performed using an Agilent 2100 Bioanalyzer. Final sequencing was conducted on an Illumina NovaSeq platform (San Diego, CA, USA).

### 2.5. Sequencing Data Assembly and Bioinformatic Analysis

According to the method described by Zhang et al. [8] and Yu et al. [9], we performed bioinformatics analysis on whole-genome sequencing data to assemble sequences of eight different influenza virus segments. Briefly, raw sequencing reads were first subjected to quality assessment and removal of adapter sequences/low-quality reads using Fastp. Quality-controlled sequences were then aligned against a host genome database using Kraken2 to eliminate host-derived sequences. De novo assembly was conducted with Megahit, followed by assembly polishing with Pilon and sequence consensus optimization using CAP3. The improved sequences served as references for reassembling high-quality reads to generate final reference-based assemblies. 514 H5N1 genomic sequences were downloaded from the GISAID database and NCBI, yielding eight datasets corresponding to the eight gene segments of H5N1. Each dataset underwent multiple sequence alignment using MUSCLE, followed by phylogenetic analysis that incorporated the H5N1 sequences obtained in our study. Maximum likelihood phylogenetic trees were constructed using FastTree, with subsequent visualization performed using the ggtree package. For spatiotemporal phylogenetic analysis, we employed Nextstrain to infer the viral evolutionary origin, mutation rate, and transmission patterns.

## 3. Results

### 3.1. Case Description and Virus Detection

During China’s 41st Antarctic Scientific Expedition, we collected samples in the vicinity of the Great Wall Station (62°12′59″ S, 58°57′52″ W) on the Fildes Peninsula, located in western King George Island of the South Shetland Islands, Antarctica.

On 7 December 2024, a critically ill, flightless thievish gull with smooth plumage but abnormal behavior was found near the Great Wall Station in China. The bird died on 10 December, and a necropsy was subsequently performed, with lung and brainstem tissues collected. Two more dead brown terns with disheveled plumage were subsequently found in the area on 25 and 26 December, and tissue samples were taken from them; a fresh fecal sample from a flying brown tern was also taken on 26 December. Real-time RT-PCR screening initially detected that these early samples were all positive for HPAI H5.

Follow-up surveillance was also conducted in 2025, and on 17 January 2025, fresh elephant seal feces were collected, and an intact submerged Antarctic fur seal carcass was found from which pharyngeal and cloacal swabs were collected. Follow-up sampling also included thieving gull feces collected on 22 January and 16 February, as well as penguin feces and a fresh fecal sample from a giant petrel collected on 13 February.

Real-time RT-PCR screening detected H5 positivity in one elephant seal fecal sample, along with H7 positivity in one fur seal sample, though subsequent retesting failed to confirm positivity in the elephant seal and fur seal specimens.

**Figure 1:**
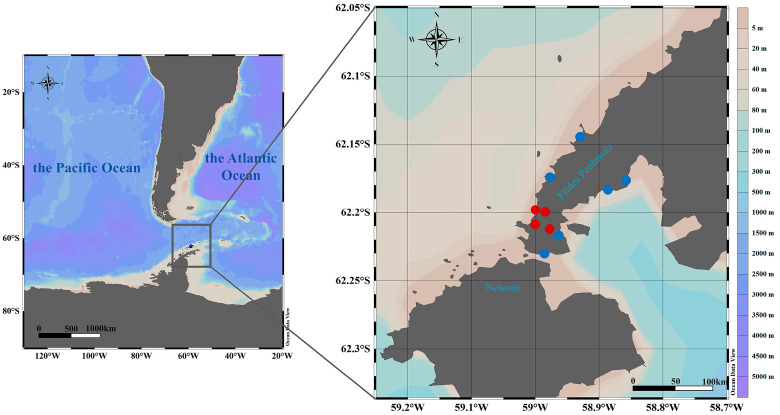
Collection sites sampled. Map of the Fildes Peninsula, South Shetland Islands, Antarctic Peninsula, showing sampling locations of animal specimens collected in this study. Complete H5N1 virus sequences were obtained from the samples marked in red.

The full chronology of sampling events and all positive species are detailed in Figure 1 and Supplementary Table S1.

### 3.2. Genomic and Phylogenetic Analysis

To investigate the potential origins, genetic characteristics, and evolution of these viruses, we performed next-generation sequencing (NGS) and phylogenetic analyses, successfully obtaining complete genome sequences for all four H5N1-positive skua samples. The H5N1 viruses identified on the Fildes Peninsula, Antarctica were formally designated as A/brown skua/Fildes Peninsula/B1/2024(H5N1), A/brown skua/Fildes Peninsula/B2/2024(H5N1), A/brown skua/Fildes Peninsula/B3/2024(H5N1), and A/brown skua/Fildes Peninsula/F4/2024(H5N1), with their complete genome sequences deposited in the Global Initiative on Sharing All Influenza Data (GISAID) under accession numbers EPI_ISL_19847535, EPI_ISL_19847536, EPI_ISL_19847538, and EPI_ISL_19847539, respectively. The genome sequences of the viruses possessed high homology with each other.

**Figure 2:**
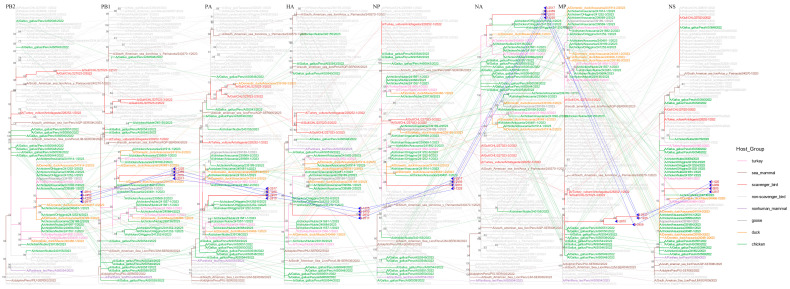
Phylogenetic incongruence analyses. Maximum likelihood trees for all eight genomic segments (PB2, PB1, PA, HA, NP, NA, MP, and NS) from equivalent strains were connected across the trees. The phylogenetic branches are color-coded to identify host species: pink lines characterize turkey, brown lines depict sea mammal, red lines exemplify scavenger bird, gray lines typify non-scavenger bird, purple lines portray nonhuman mammal, dark gray lines illustrate goose, orange lines embody duck, and green lines personify chicken. Bright blue lines represent phylogenetic connections between the genomic segments of the H5N1 strain detected on the Fildes Peninsula.

To further characterize the obtained viral strains, we retrieved 514 representative H5N1 sequences from the GISAID database for genotyping (Supplement Table S2). Subtyping based on hemagglutinin (HA) and neuraminidase (NA) protein sequences confirmed these isolates as H5N1 subtype, belonging to clade 2.3.4.4b genotype B3.2 (Figure S1). Additionally, we retrieved 1146 representative H5N1 clade 2.3.4.4b sequences from the National Center for Biotechnology Information (NCBI) for phylogenetic analysis (Supplement Table S3). Phylogenetic analysis revealed that viral sequences from the Fildes Peninsula clustered closely with H5N1 strains originating from South America, particularly demonstrating strong phylogenetic affinity with Peruvian and Chilean isolates across all genomic segments (Figure 2). These genetically similar viruses exhibit broad circulation among both avian and marine mammal populations, and South America is the predominant geographic reservoir for this viral lineage (Figures S2 and S3).

To investigate the potential introduction pathway of HPAI H5N1 to the Fildes Peninsula, we performed a discrete phylogeographic analysis using 90 closely related HA and NA sequences identified in our initial screening. The spatiotemporal reconstruction indicated Peru as the likely source country for the HPAI H5N1 virus detected on Fildes Peninsula, with transmission occurring through Chile before reaching Antarctica. Molecular dating traced the HA sequences to a Chilean strain sampled on 3 December 2022 (95% HPD: 13 November–10 December 2022), while the NA sequences originated from Chilean viruses circulating between 25 November 2022 (95% HPD: 10 November 2022–7 January 2023). This spatiotemporal reconstruction reveals a transmission pathway whereby the virus spilled over from South American poultry populations to brown skuas, subsequently reaching Fildes Peninsula’s wildlife through the birds’ natural migratory routes. (Figure S4A,B).

Comparative analysis with the reference strain A/chicken/OHiggins/241252-3/2023 revealed a 17-amino acid deletion (positions 58–74) in the stalk region of the NA protein. This deletion pattern was identical to that observed in the H5N1 strain A/brown skua/Torgersen Island/81-b82/2024 isolated from Torgersen Island but was not found in other H5N1 clade 2.3.4.4b variants. Notably, the NA gene of the Fildes Peninsula isolate harbored the substitutions E57K, V321I, and S366I, while the HA gene contained Y153H. However, continued genomic surveillance is necessary to evaluate the epidemiological significance of these findings.

## 4. Discussion

This study reports the first detection of HPAI H5N1 in brown skuas on the Fildes Peninsula, Antarctica, characterized by a unique 17-aa NA stalk deletion (58–74) currently found only in Antarctic strains. Multiple studies have documented NA stalk deletions in various influenza strains: A/Hong Kong/159/97 (H5N1) possesses a 19-aa deletion (positions 54–72) that reduces viral release capacity; A/chicken/Hubei/327/2004 (H5N1) contains a 20-aa deletion (positions 49–68); while A/Puerto Rico/8/34 (H1N1) and A/WSN/33 (H1N1) exhibit 15-aa (positions 63–77) and 16-aa (positions 57–72) deletions, respectively [10,11]. Reverse genetics studies demonstrate that NA stalk length influences viral replication kinetics; viruses with shorter stalks replicate significantly faster than their long-stalk counterparts. NA stalk length correlates with H5N1 virulence and pathogenicity; a truncated NA stalk might enhance the pathogenicity of the H5N1 subtype AIV to the mallard duck nervous system [12]. The increasing prevalence of NA stalk deletions in avian H5N1 viral isolates suggests potential adaptive advantages that may enhance cellular fitness and expand host tropism. While these mutations could significantly alter viral pathogenicity, transmissibility, and host specificity, their precise biological consequences require further experimental validation. The ecological and evolutionary significance of this Antarctic-specific deletion warrants investigation.

It should be noted that this study has limitations, including the restricted species coverage and small sample size. These constraints underscore the need for more systematic and continuous monitoring efforts to obtain more representative samples. Given the increasing human activities in Antarctica and its unique ecological significance, such surveillance is not only critical for understanding viral ecology but also essential for the early detection of potential zoonotic transmission risks.

## Data Availability

The datasets analyzed during the current study are available in the article and its supplementary material.

## Supplementary Materials

The following supporting information can be downloaded at: https://www.mdpi.com/article/10.3390/v17101365/s1. Figure S1: Phylogenetic Analysis to Confirm Genotypes; Figure S2: Phylogenetic Analysis of Geographic Origin; Figure S3: Phylogenetic Identification of Host Origin; Figure S4: Temporal–geographic–phylogenetic analysis of the HA (A) and NA (B) sequences. Table S1: Detailed information for all of the samples collected; Table S2: Detailed metadata of GISAID reference sequences used for genotyping the sequences from this study; Table S3: Detailed metadata of the reference sequences obtained from the NCBI, which were used for multiple sequence alignment and phylogenetic analysis of the sequences generated in this study.

## Abbreviations

The following abbreviations are used in this manuscript:

NGS	Next-generation sequencing
HPAI	Highly Pathogenic Human Infection with Avian Influenza
GISAID	Global Initiative on Sharing All Influenza Data
NCBI	National Center of Biotechnology Information
