# Supplementary material for: Transcontinental Spread of HPAI H5N1 from South America to Antarctica via Avian Vectors
Source: Viruses. 2025 Oct 13;17(10):1365. doi: 10.3390/v17101365 (PMC12567752; doi:10.3390/v17101365)
Supplement: Supplementary file 1 [file viruses-17-01365-s001.zip › Supplement figure legend.pdf]

Supplement figure legend

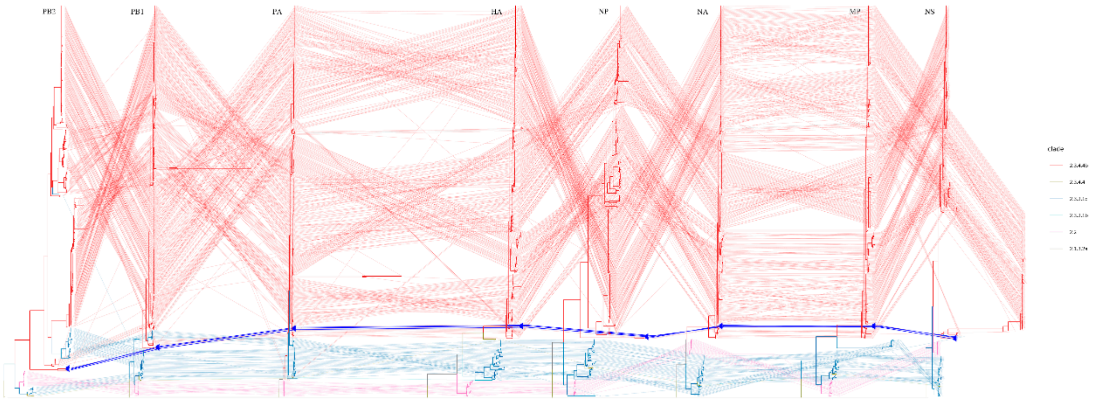

Supplement Figure S1. Phylogenetic Analysis to Confirm Genotypes. A maximum likelihood phylogenetic tree was constructed using 514 representative H5N1 sequences downloaded from the GISAID combined with sequences generated from Fildes Peninsula samples, encompassing all eight genomic segments (PB2, PB1, PA, HA, NP, NA, MP, and NS). Tip labels and connecting branches were color-coded according to the legend. Red denotes subtype 2.3.4.4b, olive green signifies 2.3.4.4, blue indicates 2.3.2.1c, cyan marks 2.3.2.1b, pink symbolizes 2.2, and light olive green represents 2.1.3.2a. Bright blue lines connect all eight genomic segments of the H5N1 strain identified on Fildes Island.

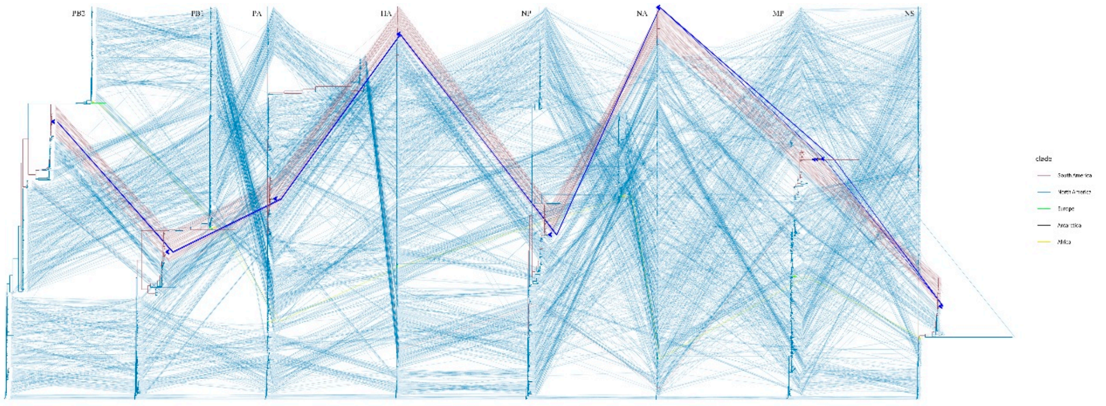

Supplement Figure S2. Phylogenetic Analysis of Geographic Origin. A maximum likelihood phylogenetic tree was constructed using 1146 representative H5N1

sequences downloaded from NCBI combined with sequences generated from Fildes Peninsula samples, encompassing all eight genomic segments (PB2, PB1, PA, HA, NP, NA, MP, and NS). The phylogenetic tree's color distinguishes geographic origins with brown indicating South America, blue symbolizing North America, green representing Europe, black denoting Antarctica, and yellow signifying Africa. Bright blue lines connect all eight genomic segments of the H5N1 strain identified on Fildes Island.

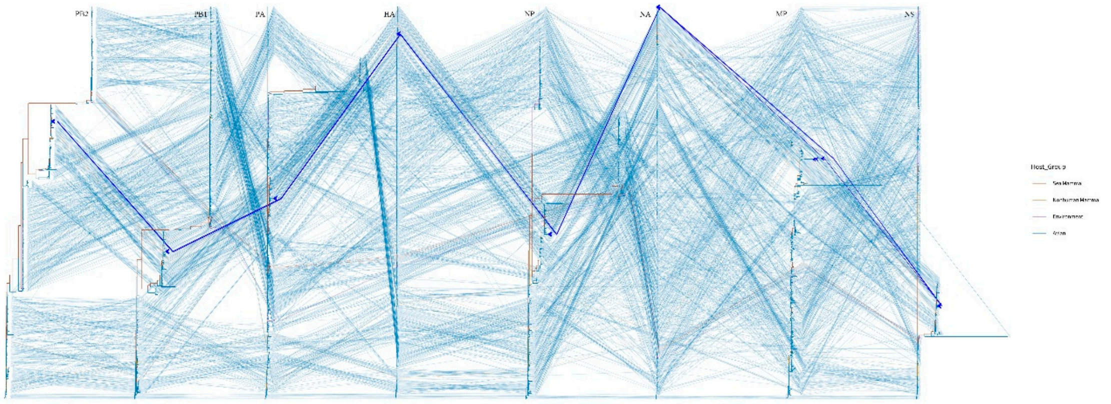

Supplement Figure S3. Phylogenetic Identification of Host Origin. A maximum likelihood phylogenetic tree was constructed using 1146 representative H5N1 sequences downloaded from NCBI combined with sequences generated from Fildes Peninsula samples, encompassing all eight genomic segments (PB2, PB1, PA, HA, NP, NA, MP, and NS). The color of the phylogenetic tree classifies host origins: brown corresponds to Sea Mammal, orange reflects Nonhuman Mammal, purple designates Environment, and sky blue characterizes Avian. Bright blue lines connect all eight genomic segments of the H5N1 strain identified on Fildes Island.

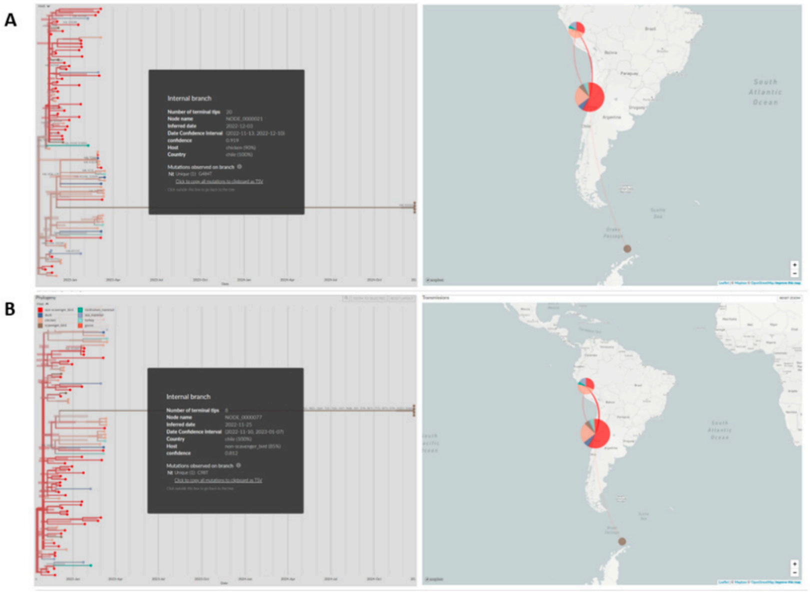

Supplement Figure S4. Temporal-geographic-phylogenetic analysis of the HA (A) and NA (B) sequences. The connecting lines indicate potential transmission routes. The color-coded legend illustrates host species classification: red squares denote non-scavenger bird, dark blue squares signify duck, light orange squares represent chicken, brown squares indicate scavenger bird, teal squares symbolize nonhuman mammal, light purple squares correspond to sea mammal, pale cyan squares stand for turkey, and orange-red squares designate goose.

Supplement Table S1. Detailed information for all the samples collected.

Supplement Table S2. Detailed metadata of GISAID reference sequences used for genotyping the sequences from this study.

Supplement Table S3. Detailed metadata of the reference sequences obtained from NCBI, which were used for multiple sequence alignment and phylogenetic analysis of the sequences generated in this study.
