# Supplementary figures and images for: Transcontinental Spread of HPAI H5N1 from South America to Antarctica via Avian Vectors
Source: Viruses. 2025 Oct 13;17(10):1365. doi: 10.3390/v17101365 (PMC12567752; doi:10.3390/v17101365)

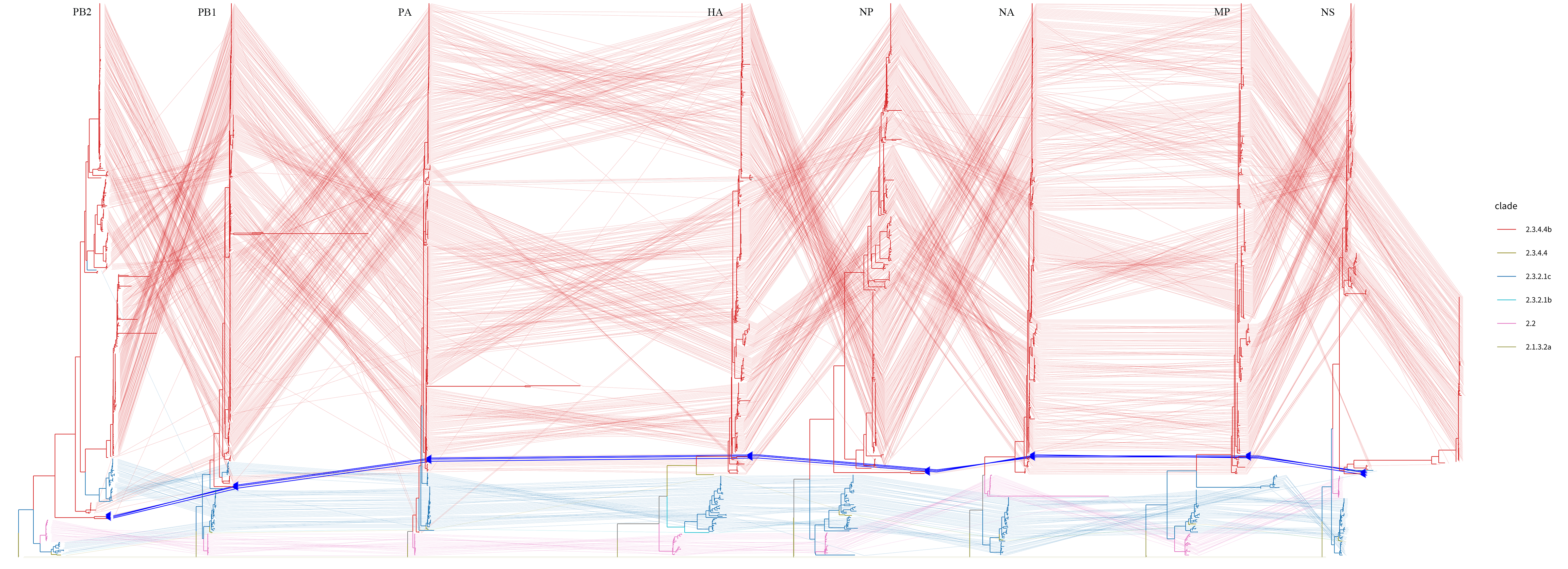

Supplement: Supplementary file 1 [file viruses-17-01365-s001.zip › Supplement Figure S1.tif]

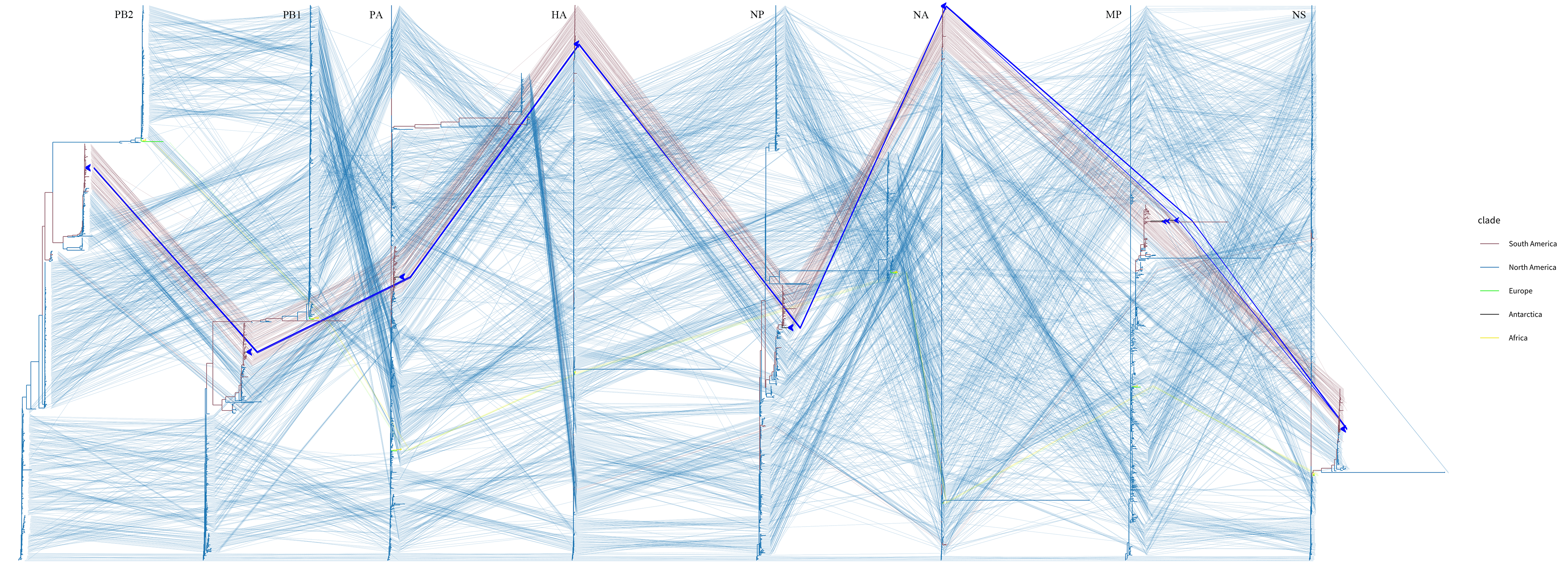

Supplement: Supplementary file 1 [file viruses-17-01365-s001.zip › Supplement Figure S2.tif]

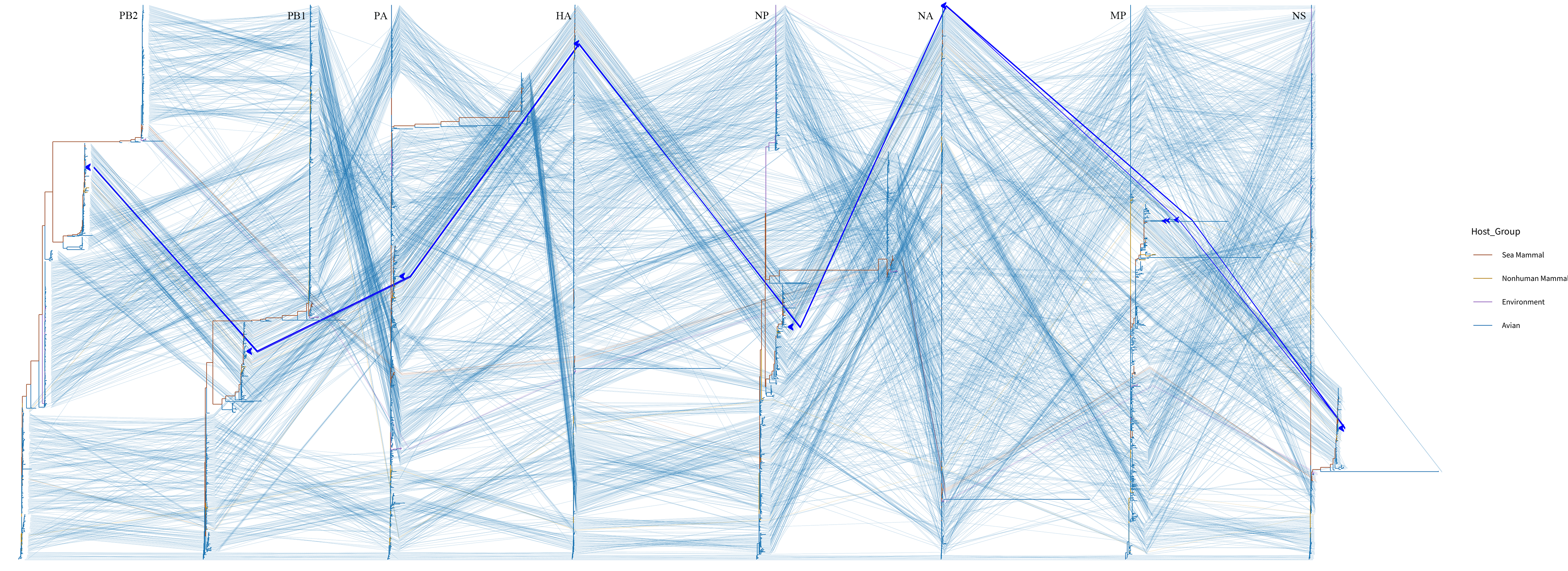

Supplement: Supplementary file 1 [file viruses-17-01365-s001.zip › Supplement Figure S3.tif]

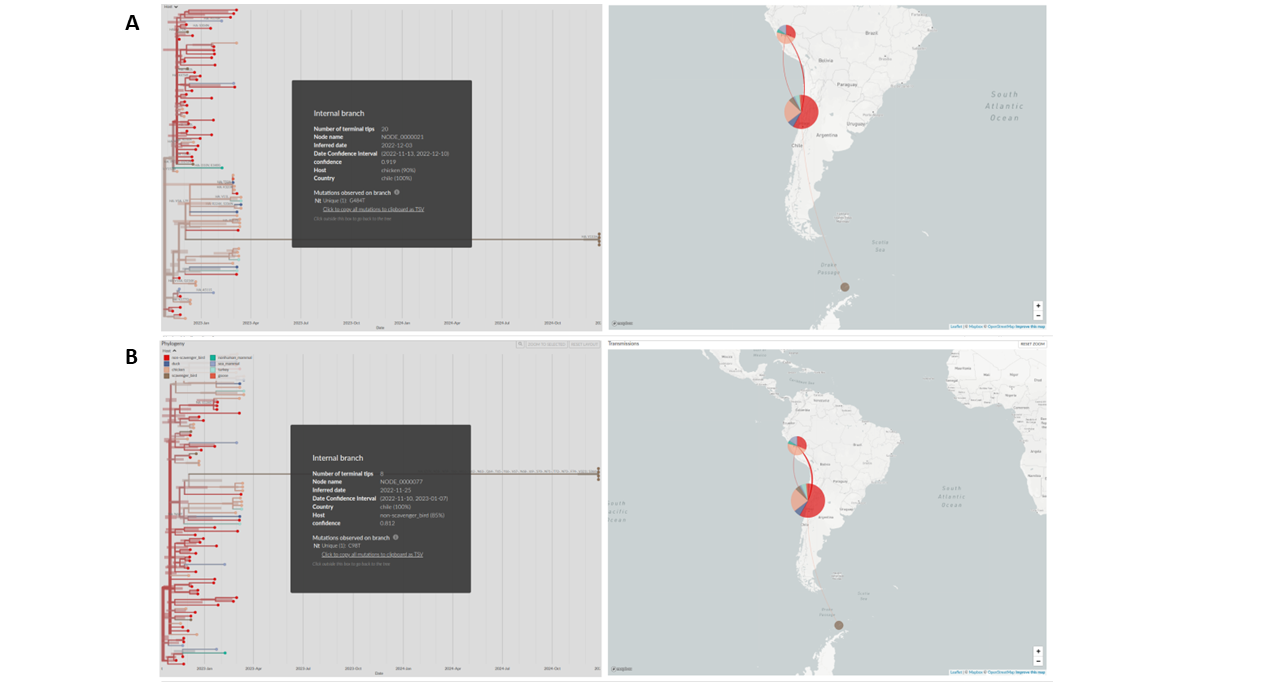

Supplement: Supplementary file 1 [file viruses-17-01365-s001.zip › Supplement Figure S4.tif]
